# Early surgical intervention for extensive nontuberculous mycobacterial pulmonary disease

**DOI:** 10.5588/ijtldopen.25.0127

**Published:** 2025-07-09

**Authors:** T.-F. Kuo, M.-R. Lee, H.-L. Huang, K.-C. Chen, M.-W. Lin, S.-W. Kuo, P.-M. Huang, H.-H. Chen, J.-Y. Wang, J.-S. Chen

**Affiliations:** ^1^ Department of Surgery, National Taiwan University Hospital and National Taiwan University College of Medicine, Taipei, Taiwan (R.O.C);; ^2^ Department of Internal Medicine, National Taiwan University Hospital and National Taiwan University College of Medicine, Taipei, Taiwan (R.O.C);; ^3^ Department of Internal Medicine, National Taiwan University Hospital Hsin-Chu Branch, Hsin-Chu, Taiwan (R.O.C);; ^4^ Division of Pulmonary and Critical Care Medicine, Department of Internal Medicine, Kaohsiung Medical University Hospital, Kaohsiung, Taiwan (R.O.C);; ^5^ Center for Liquid Biopsy and Cohort Research, Kaohsiung Medical University, Kaohsiung, Taiwan (R.O.C);; ^6^ School of Medicine, Graduate Institute of Medicine, College of Medicine, Kaohsiung Medical University, Kaohsiung, Taiwan (R.O.C);; ^7^ Institute of Epidemiology and Preventive Medicine, National Taiwan University College of Public Health, Taipei, Taiwan (R.O.C).

**Keywords:** NTM-PD, preoperative antibiotic duration, wedge resection, segmentectomy, lobectomy, pneumonectomy

## Abstract

**BACKGROUND:**

Adjuvant lung resection surgery benefits selected patients with nontuberculous mycobacterial pulmonary disease (NTM-PD); however, optimal timing remains controversial. This study evaluated surgical outcomes and prognostic factors, with a focus on the timing of surgical intervention.

**METHODS:**

This study included 41 patients with NTM-PD who underwent adjuvant lung resection surgery between January 2000 and August 2022. Data on patient characteristics, surgical procedures and postoperative outcomes were analyzed. The primary outcome, defined as freedom from unfavorable outcomes (mortality, failure to achieve sputum culture conversion, or microbiological recurrence), was estimated using the Kaplan–Meier method, with prognostic factors analyzed by Cox regression model.

**RESULTS:**

Extensive disease was observed in 35 (85%) patients. The median preoperative antibiotic duration was 3.2 months. Twenty-two (54%) patients received lobectomies, whereas 15 (37%) received wedge resections. Thirty-four (83%) achieved sputum culture conversion. The probability of being free from unfavorable outcomes within two years was 80%. Independent favorable prognostic factors included body mass index ≥ 18.5 kg/m^2^ (*p*=0.007) and early surgical intervention (preoperative antibiotic duration < 3 months, *p*=0.039). Additionally, early surgical intervention correlated with shorter operation time (*p*=0.03).

**CONCLUSIONS:**

Early surgical intervention, irrespective of the surgical approach, appeared feasible and potentially beneficial even in patients with extensive NTM-PD.

The incidence of nontuberculous mycobacterial pulmonary disease (NTM-PD) is increasing globally.^1^ Although general and supportive care is adequate for many patients, some require 1–2 years of multidrug antibiotic treatment based on clinical factors and patient preferences.^2^ However, treatment outcomes are less than satisfactory. The conversion rate is as low as 32.0–80.2% depending on the causative species.^3,4^ In addition, the adverse effects of antibiotics result in frequent treatment interruption and additional cost.^5^ To enhance infection control, adjuvant lung resection surgery is recommended for selected patients with parenchymal lung disease, including cavitary and bronchiectatic lesions.^6–9^ Surgical indications generally include structural lung disease harboring large amounts of mycobacteria, which increases the possibility of subsequent treatment failure (correction of structural lung disease), obvious and refractory respiratory symptoms, particularly recurrent hemoptysis (control of symptoms), and persistent culture positivity after 6–12 months of antibiotic treatment (failure of medical treatment).^2,10–12^ Previous studies have reported favorable outcomes and acceptable risk of complications after adjuvant surgery.^6–10,13–20^ However, the timing of surgery remains controversial. A recent meta-analysis reported preoperative antibiotic durations ranging from 2 to 24.5 months, highlighting the lack of consensus. While guidelines recommend surgery at low mycobacterial burden, they do not define the optimal timing.^2,10^

We have therefore evaluated the outcome of adjuvant lung resection surgery for NTM-PD at a tertiary referral center in Taiwan and detail the prognostic factors, focusing on timing of the intervention.

## METHODS

**Figure 1.**
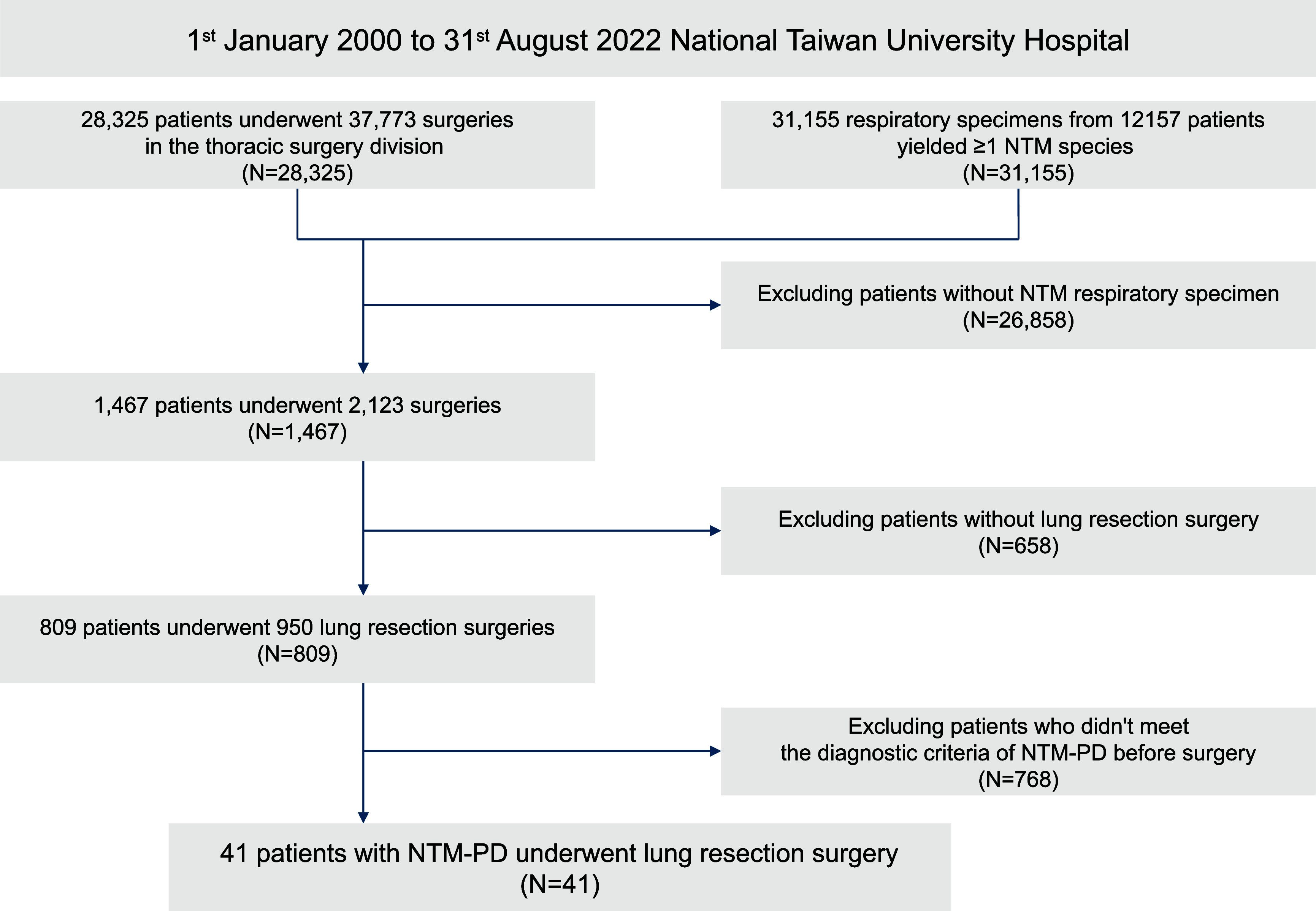
Flow chart of patient selection.

This study included patients with NTM-PD who underwent their first adjuvant lung resection surgery at the National Taiwan University Hospital between January 2000 to August 2022. The inclusion flowchart of the cohort is shown in Figure 1. During the study period, 28,325 patients underwent operations in the thoracic surgery division. After excluding patients without respiratory specimens yielding nontuberculous mycobacteria (NTM) or lung resection surgery, 809 patients were identified. Among them, 41 were diagnosed with NTM-PD per current guidelines before surgery.^2^ A total of 39 underwent unilateral surgery, while 2 underwent two-stage bilateral surgeries within 2 months.

### Primary outcome

The study’s primary endpoint was an unfavorable outcome, defined as mortality, failure to achieve sputum culture conversion, and microbiological recurrence. Patients were monitored until achieving study endpoints, or until April 30, 2023. Sputum culture conversion was defined as three consecutive negative sputum cultures without subsequent culture positivity within six months after surgery. Patients unable to cough up sputum were treated as having culture-negative sputum.^7^ Microbiological recurrence was defined as more than two positive sputum cultures within a year for patients who achieved sputum culture conversion.

### Preoperative management

Surgical indications were determined at a multidisciplinary conference including two thoracic surgeons (TFK, PMH) and three pulmonologists (HLH, MRL, JYW). The primary indications included: (1) correction of prominent structural lung disease – to eliminate the reservoir of NTM and prevent long-term complications and dismal consequences; (2) control of symptoms – for cases involving frequent or massive hemoptysis; or (3) failure of medical treatment.^2^ Additionally, the patients should have adequate pulmonary function. Both cavitary and bronchiectatic lesions were considered structural lung diseases. Failure of medical treatment was defined as persistent positive sputum culture despite effective antibiotic therapy for a total duration of six months or more, during which any single interruption should not exceed six months, progressive disease within six months under effective antibiotic therapy, or recurrent sputum culture positivity. Effective antibiotic therapy was defined as the simultaneous use of at least two guidelines-recommended drugs.^21,22^ Preoperative antibiotic duration was stratified into early and late surgical intervention groups based on prior literature and an internally derived cut-off that optimized sensitivity and specificity for predicting unfavorable outcomes.^2,23^

### Surgical technique

The operations were performed under general anesthesia with single lung ventilation. Video-assisted thoracoscopic surgery (VATS) was the preferred technique unless severe adhesion was anticipated. Planned bilateral sequential surgeries were performed for selected cases with bilateral lung lesions and were regarded as a single operation. For bilateral sequential surgeries, operative type, duration, and blood loss were defined as the summation of both procedures, while chest tube indwelling duration was defined as the average of both procedures.^24^ The primary surgical goal was the complete resection of all prominent cavitary and bronchiectatic lesions by either anatomical resection and/or multiple wedge resections. However, patients with more extensive disease or insufficient pulmonary reserve were offered selected resection of the most affected lung areas. Thus, some cavitary and bronchiectatic lesions were left and defined as remnant.^7–9^ Complete resection was defined as no remnant lesion left. During the operation, bronchi were divided and closed with staples. For patients who underwent pneumonectomy, bronchial stumps were reinforced with an intercostal muscle flap. Mediastinal lymphadenectomy was not performed. Resected specimens were sent for microbiological analysis.

### Postoperative follow-up

Postoperative complications were classified using the extended Clavien–Dindo classification.^25^ Effective antibiotic therapy continued postoperatively.

### Data collection

We obtained data on baseline patient characteristics including preoperative antibiotic duration, surgical procedures and postoperative outcomes from medical records. The 5-factor modified frailty index (mFi-5) was introduced to predict the morbidity affecting patient outcomes after lung resection surgery.^26^ Images were reviewed by one thoracic surgeon (TFK) and two pulmonologists (MRL, JYW) and classified into fibrocavitary pattern, nodular-bronchiectatic pattern, or others. Extensive disease was defined as lung lesions extending beyond a single lobe. Disease severity and extent were evaluated using a semi-quantitative CT score (maximum of 30), which comprised five categories of parenchymal abnormality: bronchiectasis (maximum score: 9), cellular bronchiolitis (maximum score: 6), cavity (maximum score: 9), nodules (maximum score: 3), and consolidation (maximum score: 3).^27^

### Statistical analyses

Statistical analyses were conducted using SPSS Statistics version 27.0 (IBM, Armonk, NY). Continuous data were presented as median (interquartile range) and categorical data as patient number (%). The internal cut-off value for early and late surgical intervention was determined using receiver operating characteristic curve analysis. The probability of being free from an unfavorable outcome was assessed using the Kaplan–Meier Method. Prognostic factors were assessed using the Cox proportional hazard regression model. Covariates with a *p*-value < 0.1 in univariate analysis were entered into stepwise multivariate analysis. A two-tailed *p*-value < 0.05 denoted statistical significance.

### Ethical statement

This retrospective cohort study was reviewed and approved by the Research Ethics Committee of the National Taiwan University Hospital, Taipei, Taiwan (202308108RINB).

## RESULTS

Clinical characteristics of the 41 patients are presented in Table 1 and Supplementary Data Table S1. Twenty-seven patients (66%) were female, and the median age was 60.0 years. All patients reported respiratory symptoms, and 17 (42%) reported constitutional symptoms. *Mycobacterium avium* complex was the most common NTM species (23 [57%]), followed by *Mycobacterium abscessus* complex (12 [29%]). Sputum acid-fast smear ≥ 2+ in 18 patients (46%). Extensive disease was observed in 35 (85%) patients, with a median semi-quantitative CT score of 13.0. The median preoperative antibiotic duration was 3.2 months, with detailed regimens provided in Supplementary Data Table S2. A total of 14 patients (34%) underwent surgery for the indication of correction of structural lung disease, 5 patients (12%) for control of symptoms and 22 (54%) for the indication of failure of medical treatment. The median preoperative antibiotic duration of each group was 1.1, 0, and 7.4 months, respectively.

**Table 1.** Baseline clinical characteristics of the 41 enrolled patients and surgical procedures.

Variable	*N* = 41**^A^**
Age (years)	60.0 [51.0–68.0]
Female sex	27 (66)
Body mass index (kg/m^2^)	19.6 [17.9–21.2]
Past or current smoker	3 (7)
Preoperative lung function	
FEV1 (% of predicted)	86.9 [67.5–97.5]
FVC (% of predicted)	85.2 [70.6–103.2]
Comorbidity	
Lung disease**^B^**	11 (27)
Previous lung surgery	4 (10)
Other systemic disease**^C^**	14 (34)
Immunosuppressant use**^D^**	4 (10)
mFi-5**^E^**	0 [0–1]
Symptom	
Respiratory symptom**^F^**	41 (100)
Constitutional symptom**^G^**	17 (42)
Radiographic features of chest CT	
Extensive disease**^H^**	35 (85)
Pattern	
Fibrocavitary	24 (58)
Nodular-bronchiectatic	15 (37)
Others	2 (5)
Semi-quantitative CT score**^I^**	13.0 [10.0–16.5]
Pathogen	
*Mycobacterium avium* complex	23 (57)
*Mycobacterium abscessus* complex	12 (29)
*Mycobacterium kansasii*	3 (7)
Other rapidly growing mycobacteria	3 (7)
Results of preoperative sputum acid-fast smear**^J^**	
Negative	9 (23)
1+	12 (31)
2+	11 (28)
3+	2 (5)
4+	5 (13)
Preoperative antibiotics duration (month)	3.2 [0.4–11.0]
Surgical indication	
Correction of structural lung disease	14 (34)
Control of symptoms	5 (12)
Failure of medical treatment	22 (54)
Type of pulmonary resection	
Pneumonectomy	2 (5)
Lobectomy	22 (54)
A single lobectomy	16 (39)
Lobectomy + segmentectomy	4 (10)
Lobectomy + wedge resections	2 (5)
Segmentectomy	2 (4)
A single segmentectomy	1 (2)
Segmentectomy + wedge resections	1 (2)
Wedge resection	15 (37)
Single	4 (10)
Multiple	11 (27)
Approach	
VATS	31 (76)
Thoracotomy	10 (24)
VATS converted to thoracotomy	2 (5)
Remnant lesions	14 (34)
Remnant cavitary lesions	8 (20)
Remnant bronchiectatic lesions	12 (29)
Operation time (min)	141 [93–249]
Estimated blood loss (mL)	25 [25–140]

Continuous data are presented as median (interquartile range), and categorical data as number (%).

A Planned bilateral sequential surgery was performed in two cases with bilateral lung lesions and was regarded as a single operation;

B Lung disease included old TB (n = 9), obstructive lung disease (n = 2), and malignancy (n = 1);

C Other systemic diseases included hypertension (n = 9), diabetes mellitus (n = 4), autoimmune diseases (n = 6) and malignancy other than lung cancer (n = 3);

D Two patients received sulfasalazine and hydroxychloroquine, one received hydroxychloroquine and one received prednisolone, mycophenolate mofetil, tacrolimus and sirolimus;

E A surrogate of frailty that may have an influence on outcome after lung resection surgery;^26^

F Respiratory symptoms included cough with sputum (n = 39), hemoptysis (n = 24), dyspnea (n = 12), and chest pain (n = 8);

G Constitutional symptoms included fever (n = 12), weight loss (n = 11), and poor appetite (n = 6);

H Extensive disease refers to the lung lesions extended beyond a single lobe;

I Semi-quantitative CT score (maximum of 30) was comprised of five categories of parenchymal abnormality, including bronchiectasis (maximum score of 9), cellular bronchiolitis (maximum score of 6), cavity (maximum score of 9), nodules (maximum score of 3), and consolidation (maximum score of 3);^27^

J Preoperative sputum acid-fast smear was not available in two patients owing to incomplete data from other hospitals.

CT = computed tomography; FEV1 = forced expiratory volume in 1 s; FVC = forced vital capacity; mFi5 = 5-factor modified frailty index; VATS = video-assisted thoracoscopic surgery.

The surgical procedures are presented in Table 1. Overall, 41 lung resection surgeries, including 2 bilateral sequential surgeries, were performed. Among these, 22 (54%) patients received lobectomies, whereas 15 (37%) received wedge resections. Resections of lesions beyond a single lobe were performed in 20 cases (49%), accounting for 57% of patients with extensive disease. Of patients who received multiple resections, 11 (55%) received multiple wedge resections. VATS was performed in 33 patients (81%), with 2 thoracotomy conversions. After surgery, 14 patients (34%) had remnant lesions, including cavitary lesions in 8 (20%) and bronchiectatic lesions in 12 (29%).

The postoperative outcomes are presented in Table 2. Complications above Clavien–Dindo grade II occurred in 8 patients (Grade II: n = 3 [7%]; Grade III: n = 4 [10%]; Grade V: n = 1 [2%]). Specifically, 1 complication (7%) occurred in the group receiving lung surgery for correcting structural lung disease, 2 (40%) in the symptom control group, and 5 (23%) in the group undergoing surgery owing to the failure of medical treatment. The patient with a grade V complication died within 30 days of the operation. Twenty-eight (72%) specimens yielded positive culture results. Sputum culture conversion was achieved in 34 (83%) patients, including 6 before surgery. Three patients (7%) were unable to provide sputum samples within 6 months and were classified as culture-negative; all had symptom resolution, radiological improvement, and no recurrence during follow-up. Among the 3 surgical indication groups, culture conversion rates were 100%, 80%, and 73%, respectively.

**Table 2.** Postoperative outcomes.

Variable	Total	Group 1**^A^**	Group 2**^B^**	Group 3**^C^**
	N = 41	N = 14	N = 5	N = 22
Complications	10 (24)	2 (14)	2 (40)	6 (27)
Air leaks > 5 days	6 (15)	2 (14)	2 (40)	2 (9)
Bronchopleural fistula	3 (7)	0 (0)	1 (20)	2 (9)
Wound dehiscence	2 (5)	0 (0)	1 (20)	1 (5)
Empyema	3 (7)	0 (0)	1 (20)	2 (9)
Pneumonia	4 (10)	1 (7)	0 (0)	3 (14)
30-day mortality	1 (2)	0 (0)	0 (0)	1 (5)
Severity of complications	8 (19)	1 (7)	2 (40)	5 (23)
Clavien–Dindo grade II**^D^**	3 (7)	1 (7)	0 (0)	2 (9)
Clavien–Dindo grade III**^E^**	4 (10)	0 (0)	2 (40)	2 (9)
Clavien–Dindo grade V**^F^**	1 (2)	0 (0)	0 (0)	1 (5)
Chest tube removal (day)	5 [3–10]			
Surgical specimen yielding NTM	28 (72)	10 (77)	4 (80)	14 (67)
Culture conversion within 6 months	34 (83)	14 (100)	4 (80)	16 (73)
Postoperative antibiotic duration (months)	11.9 [2.7–15.0]			

Continuous data are presented as median (interquartile range), and categorical data as number (%). No parameters are significantly different among the three groups.

A Group 1: Correction of structural lung disease as a surgical indication;

B Group 2: Control of symptoms as a surgical indication;

C Group 3: Failure of medical treatment as a surgical indication.

D Clavien–Dindo grade II: complications requiring drug treatment;^25^

E Clavien–Dindo grade III: complications requiring surgical, endoscopic, or radiological intervention;^25^

F Clavien–Dindo grade V: death of a patient.^25^

NTM = nontuberculous mycobacteria.

**Figure 2.**
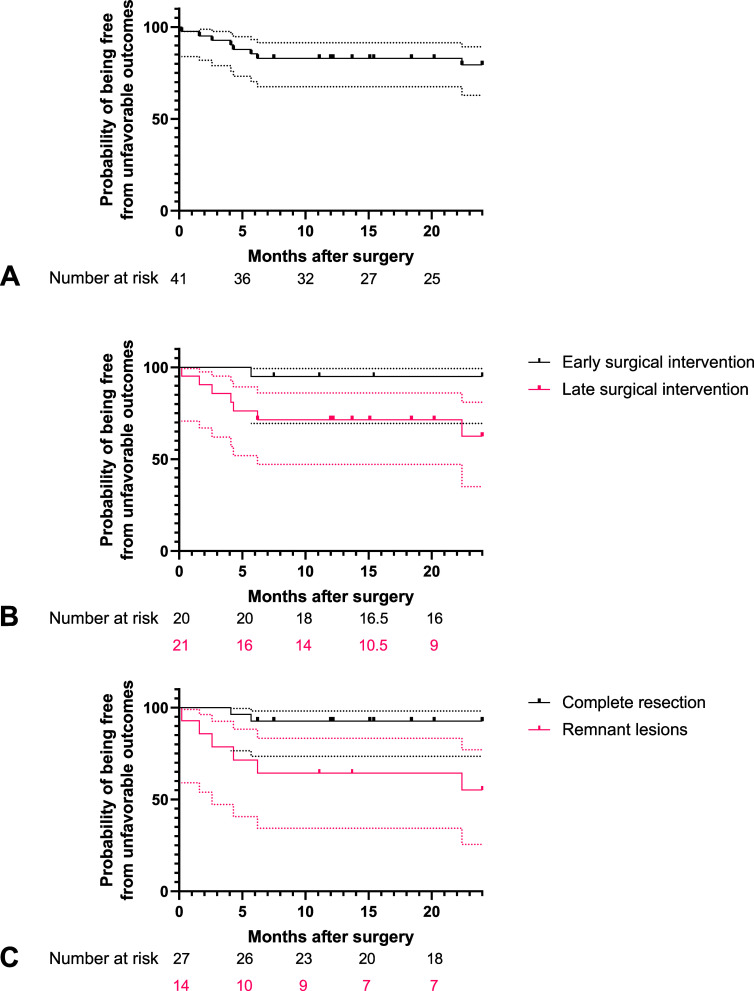
Kaplan-Meier curves for **A**: the probability of being free from unfavorable outcomes in patients after adjuvant lung resection surgery for nontuberculous mycobacterial pulmonary disease. **B**: subgroups stratified by timing of surgical intervention. **C**: subgroups stratified by completeness of resection. Subgroup analysis showing superior outcome in those receiving early surgical intervention (*p* = 0.017 by log-rank test; dotted line represents 95% confidence interval) and having complete resection of lung lesions (*p* < 0.001 by log-rank test; dotted line represents 95% confidence interval).

The curves of time-to-unfavorable outcomes are shown in Figure 2. The probability of being free from an unfavorable outcome within two years was 80% (Figure 2 Panel A). An optimal cut-off of 3.55 months was identified through receiver operating characteristic curve analysis (Supplementary Data Figure S1). Preoperative antibiotic duration was dichotomized into early (< 3 months) and late (≥ 3 months) surgical intervention and included as a covariate in the regression model. Univariate analysis revealed body mass index ≥ 18.5 kg/m^2^, early surgical intervention, and complete resection were favorable prognostic factors after operation (p=0.010, p=0.047 and p=0.018, respectively) – see Table 3. Multivariate analysis including body mass index ≥ 18.5 kg/m^2^ and early surgical intervention showed that both were independent prognostic factors, with statistical significance (*p*=0.007 and *p*=0.039, respectively) – see Supplementary Data Table S3. The probability of being free from an unfavorable outcome within 2 years was 95% and 63% in patients with early or late surgical intervention, respectively (*p*=0.017 by log-rank test; Figure 2 Panel B). The probability of being free from an unfavorable outcome within two years was 93% and 55% in patients with complete resection or those with remnant lesions, respectively (*p*<0.001 by log-rank test; Figure 2 Panel C).

**Table 3.** Univariate analysis of factors associated with an unfavorable outcome.

Variable	Univariate analysis	
	HR (95% CI)	*p*
Age ≥ 65 (years)	0.02 (0.00–8.87)	0.215
Female sex	1.55 (0.31–7.69)	0.591
Body mass index ≥ 18.5 (kg/m^2^)	0.12 (0.02–0.60)	0.010
Rapidly growing mycobacteria	1.80 (0.45–7.20)	0.406
mFi5 ≥ 2**^A^**	0.63 (0.08–5.10)	0.662
Preoperative sputum AFS ≥ 2+	98.6 (0.19–51170)	0.150
Fibrocavitary CT pattern	0.78 (0.20–3.14)	0.729
CT score ≥ 13	1.18 (0.30–4.75)	0.811
Early surgical intervention**^B^**	0.19 (0.01–0.97)	0.047
Wedge resection	1.07 (0.26–4.50)	0.924
Complete resection**^C^**	0.14 (0.03–0.71)	0.018

A A surrogate of frailty that may have an influence on outcome after lung resection surgery;^26^

B Early surgical intervention was defined as preoperative antibiotic duration < 3 months;

C Complete resection was defined as no remnant cavitary or bronchiectatic lesion left.

AFS = acid-fast smear; CI = confidence interval; CT = computed tomography; HR = hazards ratio; mFi-5 = 5-factor modified frailty index.

Compared with patients who received late surgical intervention (Supplementary Data Table S4), those received early surgical intervention had a shorter operation time (*p*=0.03), and the indications for surgery also differed significantly between groups (*p*=0.01): structural lung disease (50% vs. 19%), symptom control (20% vs. 5%), and treatment failure (30% vs. 76%), in the early and late groups, respectively.

Compared with patients with remnant lesions (Supplementary Data Table S5), those whose lung lesions were completely resected had significantly lower preoperative semi-quantitative CT scores (*p*<0.01). Additionally, the proportion of patients who received wedge resection was comparable between the two subgroups.

Compared with patients who received anatomical resection (Supplementary Data Table S6), those received wedge resection had shorter operation time (*p*=0.01) and less estimated blood loss (*p*=0.02).

## DISCUSSION

This study presents surgical outcomes of NTM-PD in a cohort that features extensive disease (85%), a relatively short preoperative antibiotic duration (3.2 months) and a high proportion of wedge resection (37%). The sputum culture conversion rate was 83%, and the 2-year probability of being free from an unfavorable outcome was 80%. Independent favorable prognostic factors after adjuvant lung resection surgery included body mass index ≥ 18.5 kg/m^2^ and early surgical intervention. Patients receiving early surgical intervention had a significantly shorter operation time than did those undergoing late surgical intervention.

Controversy remains regarding the preoperative antibiotic duration, with no definitive conclusion provided in current guidelines.^2^ Historically, the duration of preoperative antibiotics varies significantly.^10^ Some authors advocated a 16–20 months preoperative antibiotic course, whereas others recommended intensive antibiotic regimens for 2–3 months prior to surgery to maximize the reduction in bacterial burden.^11,28,29^ Yamada et al. identified longer intervals between antibiotic initiation and surgery as an independent risk factor for unfavorable outcomes, with a median preoperative antibiotic duration of 35 months.^16^ While supporting the same rationale, Ellis et al. reported that sputum mycobacterial burden decreased markedly within 3 months of treatment, suggesting potential benefits of even earlier surgical intervention.^23^ Given the concerns about the effectiveness of antibiotic penetration and acquired resistance in treating NTM-PD, early surgical correction of structural lung disease after a short intensive antibiotic treatment may improve disease control.^30^ In this study, early surgical intervention within 3 months of antibiotic initiation, guided by the findings of Ellis et al. and internal receiver operating characteristic curve analysis, was independently associated with favorable outcomes and reduced operation time.

Structural lung disease was considered a poorly penetrated area for antibiotics with a relatively large bacillary burden, leading to drug resistance and medical treatment failure.^11,31^ The concept is reinforced by the findings that remnant lesions after surgery were associated with recurrence or poor prognosis.^7–9^ Thus, extensive disease may result in refractory disease or recurrence post-surgery owing to an incomplete resection of lung lesions.^6,9^ Advances in surgical instruments and techniques have enabled wide wedge resection to remove lung parenchyma volumes comparable to segmentectomy without vessel and bronchus division,^32^ making them suitable for managing the adhesive hilar structure associated with mycobacterial infection.^11^ This may account for the shorter operation time, less blood loss, and high proportion of minimally invasive surgery in the present study. Another advantage of wedge resection is lung parenchymal preservation, making multiple lung resections in different lobes possible. This may be particularly beneficial for patients with extensive disease.

Although extensive disease (85%) with moderate to high CT scores (median: 13.0) was common in our study, our findings were mostly comparable with those reported in a recent meta-analysis on adjuvant lung resection surgery for NTM-PD, which showed sputum culture conversion rates ranging from 72–100% and recurrence rates from 0–30%.^10^ Notably, no previous studies have addressed radiological features by using semi-quantitative CT scores. However, Kim et al. reported a cohort study of 67 patients (79% female) with a median age of 57 years (60 in our cohort), a median body mass index of 20.1 kg/m^2^ (19.6 in our cohort).^9^ In that cohort, 89.6% of patients had disease involvement greater than a single lobe (85% in our cohort). This cohort was to date the most comparable patient population with our series. The median preoperative antibiotic duration was 14 months (3.2 in our cohort), and the proportion of wedge resection was 17.9% (37% in our cohort), resulting in a 71.7% sputum culture conversion rate (83% in our cohort), which is 11% less than that observed for the current cohort.

This study has some limitations. First, it involved a small cohort from a single center; although the disease severity was objectively assessed using bacteriological and radiological parameters. Second, the preoperative antibiotic regimens were not standardized due to poor medication adherence and treatment interruptions. Over-reporting of treatment duration has been a concern in previous studies.^33^ We addressed this issue by defining effective medical treatment duration as the period during which patients received guidelines-based therapy, thereby minimizing potential overestimation. Third, we defined the unfavorable outcomes as failed sputum culture conversion, microbiological recurrence, and death. The prognostic factors for each outcome in this study were impossible to be independently evaluated. Finally, given the limited number of events, we focused on univariate Cox regression. Stepwise multivariate analysis was also performed to explore potential factors, but results should be interpreted with caution.

## CONCLUSION

Early surgical intervention, irrespective of the surgical approach, appeared feasible and potentially beneficial even in patients with extensive NTM-PD.

## Supplementary Material


