# Supplementary material for: Early surgical intervention for extensive nontuberculous mycobacterial pulmonary disease
Source: IJTLD Open. 2025 Jul 9;2(7):412–9. doi: 10.5588/ijtldopen.25.0127 (PMC12248408; doi:10.5588/ijtldopen.25.0127)

Supplementary Table S1. Baseline clinical characteristics stratified by surgical indication

Variable	Total (N = 41)	Group 1[a] (N = 14)	Group 2[b] (N = 5)	Group 3[c] (N = 22)
Age (years)	60.0 [51.0–68.0]	63.5 [51.3–69.5]	64.0 [46.0–66.5]	58.5 [48.0–63.5]
Female sex	27 (66)	10 (71)	3(60)	14 (64)
Body mass index (kg·m^{-2})	19.6 [17.9–21.2]	19.7 [19.2–22.5]	20.5 [17.1–20.9]	18.8 [17.5–21.2]
Past or current smoker	3 (7)	1 (7)	0 (0)	2 (9)
Preoperative lung function				
FEV1 (% of predicted)	86.9 [67.5–97.5]	95.5 [87.5–111.6]	61.9 [46.7–87.7]	80.7 [64.5–93.8]
FVC (% of predicted)	85.2 [70.6–103.2]	100.3 [85.3–109.4]	61.1 [44.3–83.8]	81.5 [59.0–99.1]
Comorbidity				
Lung disease[d]	11 (27)	2 (14)	3 (60)	6 (27)
Previous lung surgery	4 (10)	1 (7)	1 (20)	2 (9)
Other systemic disease[e]	14 (34)	5 (36)	2 (40)	7 (32)
Immunosuppressant use[f]	4 (10)	1 (7)	0 (0)	3 (14)
mFi-5[g]	0 [0–1]	0 [0–1]	0 [0–2]	0 [0–1.3]
Symptom				
Respiratory symptom[h]	41 (100)	14 (100)	5 (100)	22 (100)
Constitutional symptom[i]	17 (42)	6 (43)	0 (0)	11 (50)
Radiographic features of chest CT				
Extensive disease[j]	35 (85)	11 (79)	5 (100)	19 (86)
Fibrocavitary pattern	24 (58)	9 (64)	2 (40)	13 (59)
Nodular-bronchiectatic pattern	15 (37)	4 (29)	3 (60)	8 (36)
Semi-quantitative CT score[k]	13.0 [10.0–16.5]	13.0 [9.8–16.3]	12.0 [11.0–19.5]	13.5 [10.0–16.3]

Pathogen				
Mycobacterium avium complex	23 (57)	11 (79)	2 (40)	10 (46)
Mycobacterium abscessus complex	12 (29)	2 (14)	2 (40)	8 (36)
Mycobacterium kansasii	3 (7)	0 (0)	1 (20)	2 (9)
Other rapidly growing mycobacteria	3 (7)	1 (7)	0 (0)	2 (9)
Results of preoperative sputum acid-fast smear[l]				
Negative	9 (23)	4 (29)	2 (40)	3 (15)
1+	12 (31)	4 (29)	2 (40)	6 (30)
2+	11 (28)	5 (36)	1 (20)	5 (25)
3+	2 (5)	0 (0)	0 (0)	2 (10)
4+	5 (13)	1 (7)	0 (0)	4 (20)
Preoperative antibiotics duration (month)	3.2 [0.4–11.0]	1.1 [0.3–3.6]	0.0 [0.0–7.5]	7.4 [2.3–13.2]
Wedge resection	15 (37)	6 (43)	1 (20)	8 (36)
Remnant lesions	14 (34)	3 (21)	1 (20)	10 (45)

Continuous data are presented as median (interquartile range) and categorical data as number (%).
CT = computed tomography; FEV1 = forced expiratory volume in 1 s; FVC = forced vital capacity; mFi-5 = 5-factor modified frailty index.
[a] Group 1: Correction of structural lung disease as a surgical indication.
[b] Group 2: Control of symptoms as a surgical indication.
[c] Group 3: Failure of medical treatment as a surgical indication.
[d] Lung disease included old tuberculosis (n = 9), obstructive lung disease (n = 2), and malignancy (n = 1).
[e] Other systemic diseases included hypertension (n = 9), diabetes mellitus (n = 4), autoimmune diseases (n = 6) and malignancy other than lung cancer (n = 3).
[f] Two patients received sulfasalazine and hydroxychloroquine, one received hydroxychloroquine and one received prednisolone, mycophenolate mofetil, tacrolimus and sirolimus.
[g] A surrogate of frailty that may have an influence on outcome after lung resection surgery.[26]
[h] Respiratory symptoms included cough with sputum (n = 39), hemoptysis (n = 24), dyspnea (n = 12), and chest pain (n = 8).

[i] Constitutional symptoms included fever (n = 12), weight loss (n = 11), and poor appetite (n = 6).
[j] Extensive disease refers to the lung lesions extended beyond a single lobe.
[k] Semi-quantitative CT score (maximum of 30) was comprised of five categories of parenchymal abnormality, including bronchiectasis (maximum score of 9), cellular bronchiolitis (maximum score of 6), cavity (maximum score of 9), nodules (maximum score of 3), and consolidation (maximum score of 3).[27]
[l] Preoperative sputum acid-fast smear was not available in two patients owing to incomplete data from other hospitals.

Supplementary Table S2. Preoperative antibiotic regimens

Preoperative antibiotic regimen	Whole period 2000–2022 (N = 41)	2000–2016 (N = 10)	2017–2022 (N = 31)
Guideline-based therapy	35 (85)	9 (90)	26 (84)
Macrolide-included regimen ≥ 3 drugs	31 (76)	8 (80)	23 (74)
Macrolide-included regimen = 2 drugs	3 (7)	1 (10)	2 (7)
Non-macrolide-included regimen ≥ 3 drugs	1 (2)	0	1 (3)
Non-guideline-based therapy	6 (15)	1 (10)	5 (16)
Aminoglycoside injection	23 (56)	4 (40)	19 (61)

Categorical data as number (%). The proportion of antibiotic therapy and the rate of aminoglycoside injection did not differ significantly between 2000–2016 and 2017–2022.

Supplementary Table S3. Multivariate analysis of factors associated with an unfavorable outcome

Variable	Multivariate analysis	
	HR (95% CI)	*p*
Body mass index ≥ 18.5 (kg·m^{-2})	0.11 (0.02–0.55)	0.007
Early surgical intervention[a]	0.11 (0.01–0.90)	0.039

CI = confidence interval; HR = hazards ratio.
[a] Early surgical intervention was defined as preoperative antibiotic duration < 3 months.

Supplementary Table S4. Comparison between early intervention (preoperative antibiotics duration < 3 months) and late intervention (preoperative antibiotics duration ≥ 3 months)

Variable	Early intervention (N = 20)	Late intervention (N = 21)	*p*
Age (years)	63 [52–69]	59 [45–65]	0.23
Female sex	15 (75)	12 (57)	0.33
Body-mass index (kg·m^{-2})	19.6 [18.0–21.1]	19.4 [17.8–21.5]	0.98
Preoperative lung function			
FEV1 (% of predicted)	86.3 [67.5–108.4]	88.5 [67.4–96.0]	0.78
FVC (% of predicted)	84.1 [70.6–105.6]	86.3 [61.6–103.1]	0.90
mFi-5[a]	0 [0–1]	0 [0–1]	0.69
Preoperative sputum smear ≥ 2+	7 (35)	11 (58)	0.20
CT score ≥ 13[b]	10 (50)	10 (48)	>0.99
Extensive disease	18 (90)	17 (81)	0.66
Surgical indication			0.01
Correction of structural lung disease	10 (50)	4 (19)	
Control of symptoms	4 (20)	1 (5)	
Failure of medical treatment	6 (30)	16 (76)	
Operation time (min)	105 [85–161]	172 [107–277]	0.03[c]
Estimated blood loss (mL)	25 [25–100]	50 [25–325]	0.09
Wedge resection	7 (35)	8 (38)	1.00
Remnant lesions	6 (30)	8 (38)	0.74
Sputum culture conversion	19 (95)	15 (71)	0.09
After surgery	15 (75)	13 (62)	0.51
Chest tube removal (day)	5 [2–9]	5 [3–10]	0.88
Morbidity and Mortality	4 (20)	6 (29)	0.72

Continuous data are presented as median (interquartile range) and categorical data as number (%). *p* values are from the Mann–Whitney U test, Chi-squard test and Fisher's exact test.

CT = computed tomography; FEV1 = forced expiratory volume in 1 s; FVC = forced vital capacity; mFi-5 = 5-factor modified frailty index.

[a] surrogate of frailty that may make an influence on outcomes after lung resection surgery.[26]

[b] Semi-quantitative CT score (maximum of 30) was comprised of five categories of parenchymal abnormality including bronchiectasis (maximum score of 9), cellular bronchiolitis (maximum score of 6), cavity (maximum score of 9), nodules (maximum score of 3), and consolidation (maximum score of 3).[27]

[c] Intergroup difference is -57.5 (95% confidence interval: -134–-4), calculated using the Hodges–Lehmann estimation.

Supplementary Table S5. Comparison between patients with complete resection and those with remnant lesions

Variable	Complete resection (N = 27)	Remnant lesions (N = 14)	*p*
Age (years)	60 [46–66]	59 [51–70]	0.58
Female sex	16 (59)	11 (79)	0.30
Body-mass index (kg·m^{-2})	20.3 [18.4–21.3]	18.9 [17.2–20.6]	0.12
Preoperative lung function			
FEV1 (% of predicted)	92.3 [77.7–102.9]	73.9 [51.4–90.9]	0.06
FVC (% of predicted)	88.9 [79.5–104.4]	77.8 [53.5–88.3]	0.08
mFi-5[a]	0 [0–1]	0.5 [0–3.3]	0.31
Preoperative sputum smear ≥ 2+	9 (35)	9 (69)	0.09
CT score ≥ 13[b]	9 (33)	11 (79)	<0.01
Extensive disease	21 (78)	14 (100)	0.08
Operation time (min)	98 [154–245]	112 [86–257]	0.58
Estimated blood loss (mL)	25 [25–150]	25 [25–125]	0.67
Wedge resection	9 (33)	6 (43)	0.73
Sputum culture conversion	25 (93)	9 (64)	0.04
After surgery	21 (78)	7 (50)	0.09
Chest tube removal (day)	5 [2–10]	5 [3–8]	0.96
Morbidity and mortality	7 (26)	3 (21)	1.00

Continuous data are presented as median (interquartile range) and categorical data as number (%). *p* values are from the Mann–Whitney U test and Fisher's exact test.
CT = computed tomography; FEV1 = forced expiratory volume in 1 s; FVC = forced vital capacity; mFi-5 = 5-factor modified frailty index.
[a] surrogate of frailty that may make an influence on outcomes after lung resection surgery.[26]
[b] Semi-quantitative CT score (maximum of 30) was comprised of five categories of parenchymal abnormality including bronchiectasis (maximum score of 9), cellular bronchiolitis (maximum score of 6), cavity (maximum score of 9), nodules (maximum score of 3), and consolidation (maximum score of 3).[27]

Supplementary Table S6. Comparison between patients receiving anatomical resection and those receiving wedge resection

Variable	Anatomical resection (N = 26)	Wedge resection (N = 15)	*p*
Age (years)	61 [55–68]	51 [38–69]	0.07
Female sex	17 (65)	10 (67)	>0.99
Body-mass index (kg·m^{-2})	19.6 [18.3–21.1]	19.4 [17.7–21.4]	0.76
Preoperative lung function			
FEV1 (% of predicted)	86.7 [67.9–103.0]	92.1 [66.7–97.9]	0.97
FVC (% of predicted)	84.5 [66.6–104.1]	89.6 [78.3–102.4]	0.91
mFi-5[a]	1 [0–1]	0 [0–1.3]	0.66
Preoperative sputum smear ≥ 2+	11 (44)	7 (50)	0.75
CT score ≥ 13[b]	12 (46)	8 (53)	0.75
Extensive disease	22 (85)	13 (87)	1.00
Operation time (min)	167 [110–274]	101 [86–152]	0.01[c]
Estimated blood loss (mL)	100 [25–263]	25 [25–25]	0.02[d]
Remnant lesions	8 (31)	6 (40)	0.73
Sputum culture conversion	21 (81)	13 (87)	1.00
After surgery	18 (69)	10 (67)	1.00
Chest tube removal (day)	5.0 [3–11]	5.0 [1–10]	0.66
Morbidity and Mortality	7 (27)	3 (20)	0.72

Continuous data are presented as median (interquartile range) and categorical data as number (%). *p* values are from the Mann–Whitney U test and Fisher's exact test.
CT = computed tomography; FEV1 = forced expiratory volume in 1 s; FVC = forced vital capacity; mFi-5 = 5-factor modified frailty index.
[a] surrogate of frailty that may make an influence on outcomes after lung resection surgery.[26]
[b] Semi-quantitative CT score (maximum of 30) was comprised of five categories of parenchymal abnormality including bronchiectasis (maximum score of 9), cellular bronchiolitis (maximum score of 6), cavity (maximum score of 9), nodules (maximum score of 3), and consolidation (maximum score of 3).[27]
[c] Intergroup difference is 60.5 (95% confidence interval: 12–143), calculated using the Hodges–Lehmann estimation.
[d] Intergroup difference is 25.0 (95% confidence interval: 0–80), calculated using the Hodges–Lehmann estimation.

Supplementary Figure S1. Receiver operating characteristic curve analysis showed the trade-off between sensitivity and specificity. Preoperative antibiotic duration had good discriminatory ability for identifying patients at risk of an unfavorable outcome, with an area under the ROC curve of 0.782 (95% confidence interval, 0.572–0.993). The optimal cut-off value for early intervention was 3.55 months.

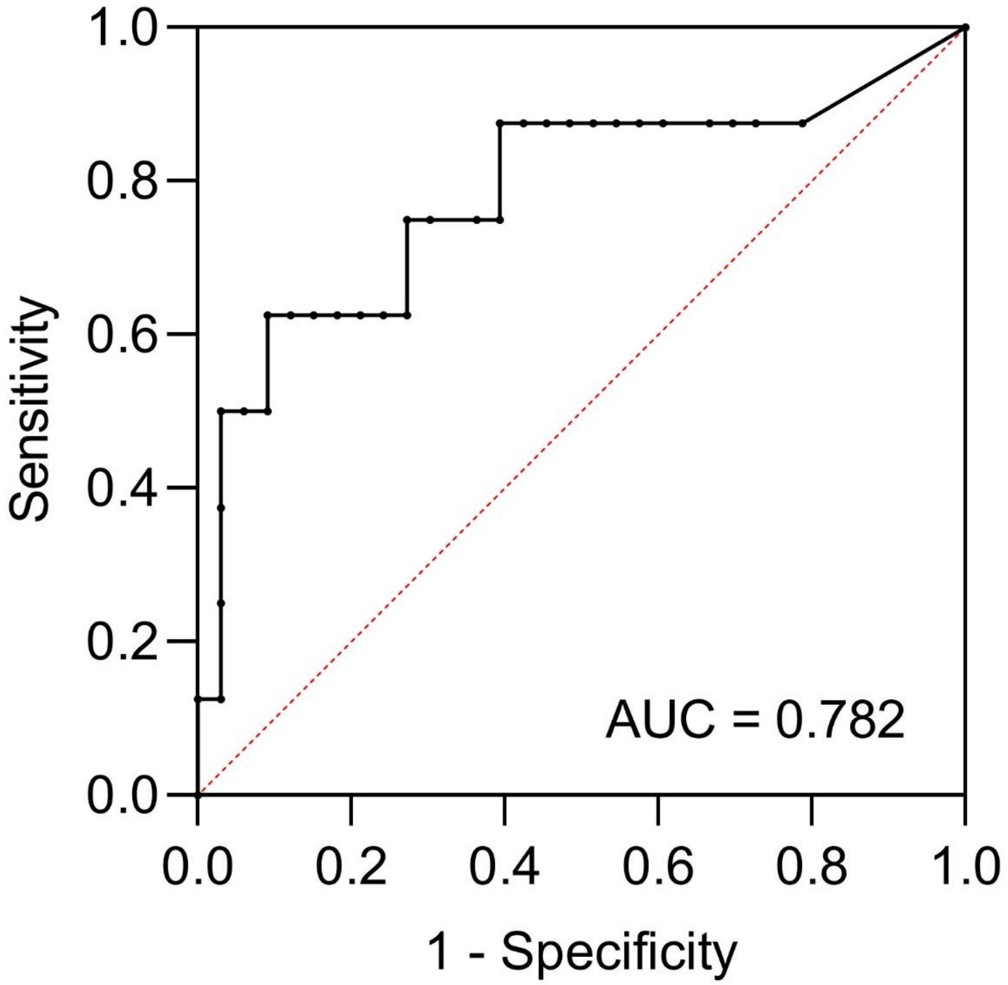

Supplement: Supplementary file 1 [file ijtldopen25-0127_supplementarydata1.pdf]
